# Efficacy of Non-Invasive Monopolar Radiofrequency for Treating Genitourinary Syndrome of Menopause: A Prospective Pilot Study

**DOI:** 10.3390/clinpract15080155

**Published:** 2025-08-20

**Authors:** Mariachiara Palucci, Marta Barba, Alice Cola, Clarissa Costa, Desirèe De Vicari, Matteo Frigerio

**Affiliations:** Department of Gynecology, Fondazione IRCCS San Gerardo dei Tintori, University of Milano-Bicocca, 20900 Monza, Italy; m.barba8792@gmail.com (M.B.); alice.cola1@gmail.com (A.C.); c.costa14@campus.unimib.it (C.C.); d.devicari@campus.unimib.it (D.D.V.); frigerio86@gmail.com (M.F.)

**Keywords:** radiofrequency, genitourinary syndrome of menopause, sexual dysfunction, vulvovaginal atrophy

## Abstract

**Introduction**: The decline of serum estrogen in postmenopausal women leads to several changes in the vulvovaginal and vesicourethral areas, resulting in the genitourinary syndrome of menopause (GSM), characterized by bothersome symptoms such as vaginal atrophy, lack of lubrication, dyspareunia, urgency, dysuria, and recurrent urinary tract infections. Nevertheless, this condition could also be experienced by younger women affected by hormone-dependent tumors. Although topical estrogens are considered “the gold standard”, hormonal treatments cannot be indicated in cancer survivors. As a result, energy-based devices using radiofrequency and laser technologies have emerged as alternative options. This prospective study aimed to evaluate the benefits of non-invasive monopolar radiofrequency (RF) in women affected by GSM who have contraindications to, did not respond to, or declined local estrogen therapy. **Methods**: The patients underwent five weekly sessions of second-generation monopolar RF. At baseline and at the fifth session, two validated questionnaires were administered to the patients: the Visual Analogue Scale (VAS) and the Female Sexual Function Index (FSFI-19). On the other hand, the vaginal mucosa status was evaluated by clinicians through the Vaginal Health Index (VHI). At the end of the cycle, the Patient Global Impression of Improvement (PGI-I) questionnaire was collected. **Results**: Based on 44 patients who completed five sessions of radiofrequency, a significant improvement was observed in sexual function according to the FSFI scale (22.9 vs. 38.6; *p* < 0.001) and in VVA atrophy symptoms, as documented by the VAS score (223 vs. 125; *p* < 0.001). The mean VHI score increased by 3 points (*p* < 0.001). Moreover, according to PGI-I, 96% of patients reported a perceived improvement (PGI-I score ≤ 3). **Conclusions**: Radiofrequency could provide an innovative and safe therapeutic approach for patients suffering from GSM and VVA, especially when hormonal strategies are unsuitable.

## 1. Introduction

The genitourinary syndrome of menopause (GSM) is a relative new terminology, formally adopted in 2014 through a consensus between the International Society for the Study of Women’s Sexual Health and the North American Menopause Society in order to better describe a condition previously named vulvovaginal atrophy (VVA), atrophic vaginitis, or urogenital atrophy [1]. GSM is a more accurate and inclusive term which embraces a wide spectrum of genital, urological, and sexual symptoms associated with the decline of serum estrogens and other sex steroids [2].

The syndrome occurs in some manner in approximately 15% of premenopausal women due to hypoestrogenic state (lactation, breast cancer treatments, pelvic surgery, or radiotherapy) and 40–50% of postmenopausal women [3]. This condition is often underdiagnosed because patients feel embarrassed to seek help or view it as a natural part of aging [4]. In fact, the VIVA study showed that although 45% of postmenopausal women reported vaginal symptoms, only 4% identified them as related to menopause [5].

Due to their shared embryological origin, the genitalia and lower urinary tract both rely on estrogen receptors, specifically estrogen receptor α, which is present in both pre- and postmenopausal women, and estrogen receptor β, which is active only during the fertile years [6].

Physiologically, tissue atrophy results from several changes: decreased expression of estrogen receptors, reduced vascularization [7], thinning of the vaginal epithelium, collagen degradation and loss of elasticity, decreased hyaluronic acid content, and alterations in the quality and quantity of vaginal secretions [8]. Clinically, these changes cause symptoms like tissue fragility, recurrent infections, irritation, dryness, discomfort, pain during intercourse, urinary leakage, and a significant decline in quality of life. If untreated, these symptoms usually worsen over time [1,9,10,11,12]. Additionally, vaginal relaxation syndrome (VRS) may develop, causing genital laxity, aesthetic concerns, stress urinary incontinence, and reduced sensation during coitus [13].

Furthermore, estrogen receptors located in the bladder trigone and urethra are believed to play a critical role in modulating sensory perception during bladder filling by raising the sensory threshold. In the absence of a sufficient estrogen level, this threshold is lowered, resulting in heightened bladder sensitivity and reduced urethral closure pressure, as well as diminished Valsalva leak-point pressure. These alterations collectively contribute to the development of urinary urgency [14] and stress urinary incontinence [15].

Optimal management of GSM requires a personalized strategy based on symptom profile, medical history, lifestyle, and a clear assessment of the risks and benefits of estrogen therapy (ET). The first-line treatment includes non-hormonal therapies, such as lubricants and moisturizers, while local estrogen products are considered the “gold standard”. Newer hormonal therapeutic strategies may involve selective estrogen receptor modulators (SERMs) or intravaginal dehydroepiandrosterone (DHEA, Prasterone); however, despite the proven benefits on VVA symptoms and on dyspareunia, the safety in cancer survivors remains controversial, leading to a significant concern [16]. Typically, in this specific population—treated with pelvic irradiation, chemotherapy, or prolonged hormonal therapy—symptoms can be more severe and persistent; therefore, standard treatment protocols using topical hyaluronic acid and lubricants may be insufficient [17].

Recently, energy-based devices have been developed as an innovative alternative for such scenarios [18]. Existing literature comprises a substantial number of clinical studies evaluating the efficacy and safety of vaginal laser therapies—both ablative and non-ablative—for the treatment of VVA, with the majority focusing on the application of micro-ablative fractional CO_2_ lasers and non-ablative erbium-doped yttrium aluminum garnet (Er: YAG) lasers [19]. Despite the dissimilarities between these two types of lasers, their primary effect is believed to involve neocollagenesis, elastogenesis, and neoangiogenesis, which stimulate tissue remodeling and rejuvenation [20]. Although there are well-known benefits, the ablative laser techniques, because of their mechanism of action, may lead to adverse effects, such as scarring, infections, pigmentation changes, and inflammation [21]. Non-ablative technologies have been proposed in an effort to minimize complications, reduce perioperative pain, and accelerate healing by inducing controlled dermal injury without ablating the epidermis [22]. In this context, alongside the previously mentioned non-ablative vaginal lasers, radiofrequency (RF) technology is also emerging as a therapeutic option. A comparative overview of the key features of various devices is presented in Table 1.

Capacitive radiofrequency (RF) is widely used in dermatology and aesthetic medicine because of beneficial effects in the treatment of skin laxity, fine lines, wrinkles, and cellulite. It promotes skin tightening and rejuvenation without damaging the epidermis, making it a safe and effective option for patients seeking non-surgical skin treatments [23,24].

Consequently, the mechanism of RF seems to be suitable for GSM/VVA and has been adopted for gynecological applications. Unlike lasers, which convert light into heat through the selective absorption of light by superficial structures or specific chromophores via the mechanism of selective photothermolysis, RF generates heat through tissue electrical resistance, which transforms electrical current into thermal energy within the deeper layers of the dermis while simultaneously preserving the integrity of the epidermis [25]. Specifically, the isolated (capacitive) elements concentrate the energy load on the nearby tissues in order to achieve greater selectivity toward the target of interest.

Although numerous studies have investigated the effectiveness of various laser therapies for the treatment of VVA, clinical evidence supporting the use of RF for GSM remains limited and significantly less robust. Moreover, there is a lack of standardized and well-defined protocols for RF application in this context, making it difficult to compare outcomes across studies and establish clear clinical guidelines. In light of this important gap in literature, our study aims to evaluate the efficacy and safety of outpatient monopolar capacitive RF treatment in women with moderate to severe GSM symptoms who have contraindications to, did not respond to, or refused local estrogen therapy.

## 2. Materials and Methods

This prospective pilot study was conducted with prior approval from the local ethics committee (approval NO. 54, 23 January 2025; protocol code GSM-RF). Participant recruitment occurred between January and June 2025 from the gynecology outpatients of a tertiary-level urogynecology center (Fondazione IRCCS San Gerardo dei Tintori in Monza, Italy). Prior to referral for treatment, all patients underwent a urogynecological evaluation, including a detailed assessment of GSM symptoms and duration, previous hormonal treatments received or any contraindications to hormonal therapy, and a clinical examination of the external genitalia. Patients were deemed eligible if they were 18 years or older, had experienced at least two of the previously mentioned GSM symptoms for a minimum duration of one year, and either had contraindications to or refused local estrogen therapy. All patients voluntarily consented to participate in the study and signed written informed consent during their initial session.

**Figure 1:**
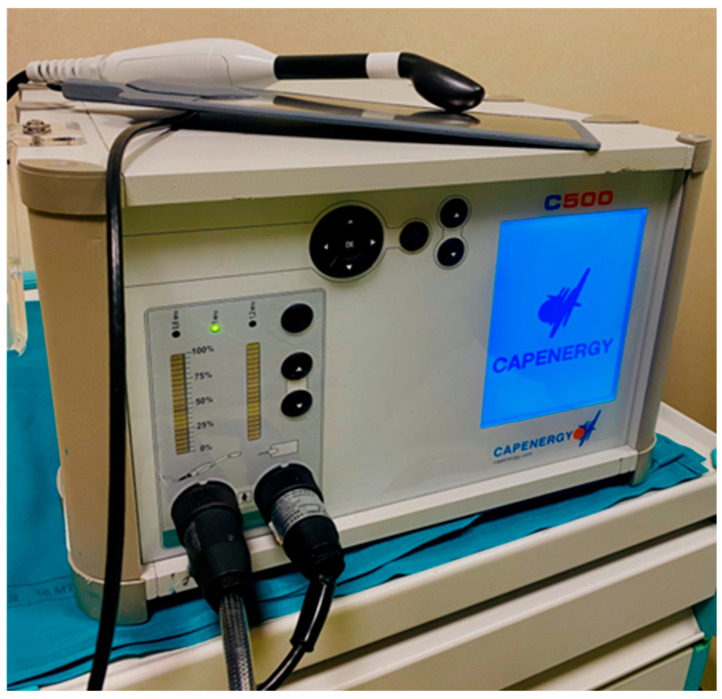
C500 Urogyne device (Capenergy, Barcelona, España).

The treatment cycle consisted of a single weekly radiofrequency session, lasting 20 min, for five consecutive weeks. The device used was a monopolar capacitive intravaginal radiofrequency system “C500 Urogyne (Capenergy, Medswiss)”, as shown in Figure 1, consisting of two electrodes: an active intracavitary electrode inserted into the vagina and a dispersive passive electrode positioned in the lumbosacral area. A major advantage of this device is that it features a second-generation, high-power, non-ablative radiofrequency system equipped with dual temperature sensing technology, providing greater safety, precision, and control during treatment. The probe (active electrode) was covered with a non-lubricated condom, and a water-based gel was applied to facilitate movement over the external genitalia and insertion into the vagina without causing discomfort. The two adjustable parameters were power and frequency, which were regulated to guarantee that the temperature remained below 39 °C on the external genitalia and below 42 °C during intravaginal application. Throughout the session, the patient was asked to report her perception of the heat—ensuring it was never burning but rather a pleasant warmth—as well as to indicate any areas where pain or discomfort was experienced.

At baseline, participants were evaluated using the Vaginal Health Index (VHI), which includes five parameters: elasticity, fluid volume, pH, epithelial integrity, and moisture. The total score ranges from 5 to 25, with a cutoff of 15; scores below this threshold are indicative of atrophic vaginitis [26]. The VHI is considered a semi-objective measure, as four out of the five components—excluding vaginal pH, which is measured with a pH indicator strip—are subject to the clinician’s judgment. In addition, the severities of vulvovaginal atrophy (VVA) symptoms, including vaginal burning, itching, dryness, dyspareunia, and dysuria, were assessed using a 10 cm Visual Analog Scale (VAS), where 0 represents “no symptom” and 100 represents “worst imaginable symptom” [27]. The total VAS score represents the sum of the scores assigned to the five individual symptoms. Sexual function was evaluated using the 19-item Female Sexual Function Index (FSFI-19) questionnaire [28]. This self-reported instrument uses a 5-point Likert scale and covers six domains: sexual desire, arousal, lubrication, orgasm, pain, and satisfaction. A total FSFI score of 26.5 is commonly used as the cutoff to differentiate between women with and without sexual dysfunction.

At the fifth treatment session, the Vaginal Health Index (VHI) was reassessed by the same clinician who performed the pre-treatment evaluation. In the same way, the severities of VVA symptoms and sexual function were re-evaluated using the VAS score and FSFI-19 questionnaire, respectively. Patients also completed the Patient Global Impression of Improvement (PGI-I) questionnaire, a 7-point Likert scale designed to assess perceived changes in their condition compared to baseline. This tool enables clinicians to analyze the overall degree of improvement or deterioration, with response options ranging as follows: 1—very much improved, 2—much improved, 3—minimally improved, 4—no change, 5—minimally worse, 6—much worse, and 7—very much worse.

All patients were closely monitored throughout the treatment period for any adverse effects. Anonymized data were prospectively collected by the investigators. Statistical analysis was conducted using Jamovi (software Version 2.3) [29]. Continuous variables are presented as means ± standard deviations, while categorical variables are expressed as absolute or relative frequencies. Pre- and post-treatment outcomes, both objective and subjective, were compared using paired *t*-tests, with statistical significance defined as *p*-value < 0.05.

## 3. Results

A total of 48 patients were enrolled. The average age was 53.9, with the youngest patient being 34 years old and the oldest 75 years old. A total of 27 out of 48 women (56.3%) had at least one vaginal delivery, while 26 (54.2%) had undergone pelvic surgeries. Furthermore, 28 (58.3%) were oncology patients with a previous diagnosis of hormone-dependent tumors. All patients were in menopause, either spontaneous or pharmacologically/surgically induced, due to their tumor-related condition.

The demographic characteristics of the population are summarized in Table 2.

A total of 45 patients (93.8%) successfully completed all five planned sessions of radiofrequency. Two patients underwent three sessions, and one patient only two, due to the high frequency of the treatment and personal commitments that made weekly hospital visits unfeasible. No patients discontinued the treatment due to procedural intolerance. One patient was lost to follow-up because she never returned the post-treatment questionnaires. Therefore, the reported results are based on 44 women for whom we have both pre- and post-treatment comparisons. The mean scores of the pre- and post-treatment questionnaires are presented in Table 3. We observed a significant improvement in sexual function, as demonstrated by both the total FSFI-19 score (22.9 vs. 38.6; *p* < 0.001) and the individual domains. Similarly, the severity of GSM symptoms significantly decreased according to the total VAS score (from 223 to 125; *p* < 0.001), with particularly notable improvements observed in dyspareunia, dryness, and itching (*p* < 0.001). Additionally, clinical quality of the vaginal mucosa and external genitalia improved, as evidenced by a 3-point increase in the VHI from 13.5 to 16.5 (*p* < 0.001). At the end of the treatment, approximately 95.5% of patients reported a PGI-I score of less than 3, indicating a subjective improvement in symptoms.

The treatment was well tolerated by all patients, and no adverse effects were reported.

## 4. Discussion

Genitourinary syndrome of menopause (GSM) and vulvovaginal atrophy (VVA) represent significant, yet frequently underestimated, health concerns for aging women, particularly in light of the global increase in life expectancy [30]. Despite the high prevalence and impact of these conditions on quality of life, they are often underdiagnosed or misinterpreted as natural consequences of aging or side effects of oncologic therapies. This clinical under-recognition may stem from both patient hesitancy in reporting symptoms and insufficient awareness among healthcare providers regarding the chronic and progressive nature of these disorders [5,31]. VVA, a central component of GSM, is a chronic degenerative condition resulting from the estrogen deficiency characteristic of menopause. The prevalent signs include atrophic changes affecting both the external and internal female genitalia, with regression and thinning of the labia minora, narrowing of the vaginal introitus, and prominence of the urethral meatus [32]. On a histological level, characteristic tissue alterations manifest as thinning of the stratified squamous epithelium, reduced glycogen content within epithelial cells, and a loss of vascularization and dermal papillae [33]. Without proper treatment, the consequent symptoms, such as vaginal dryness, dyspareunia, burning, and urinary discomfort, typically persist or worsen over time [34]. In cancer survivors, especially those who have undergone oophorectomy or received gonadotoxic therapies, these symptoms can manifest earlier and with greater intensity due to the abrupt hormonal withdrawal [35,36]. The limited evidence on the safety of vaginal hormone therapies in this specific population has led many women to avoid treatment or opt for only non-hormonal alternatives, potentially compromising their quality of life and intimate relationships [37]. In this sense, first-line options typically include water- or silicone-based vaginal lubricants, vaginal moisturizers, or herbal remedies [38]. Although these products can offer temporary relief of vaginal dryness and of pain during sexual activity, they do not target the underlying tissue changes associated with estrogen deprivation and, therefore, lack of long-term efficacy [39]. Alongside the traditional treatments, energy-based devices emerged in recent years as a promising non-hormonal strategy for the management of VVA. Laser therapy works by stimulating mechanisms to repair, grow, and heal the tissues, resulting in an increase in capillary density and connective tissue remodeling, which is achieved through different modes of action, depending on whether the laser used is ablative or non-ablative [40]. The most popular micro-fractional CO_2_ lasers exert an ablative effect on tissues by creating microscopic columns of thermal injury into the deeper tissues, preserving the superficial tissue, which subsequently stimulates fibroblast activation and collagen production [40,41]. Furthermore, in order to overcome the main adverse effects of ablative lasers, such as discomfort during treatment, edema, or pigmentation changes [40], non-ablative laser and radiofrequency (RF) have been widely used. RF devices emit concentrated electromagnetic waves that generate heat in the deeper tissue layers without affecting melanin. Thanks to the resistance provided by the tissue impedance, this thermal energy promotes collagen contraction, stimulates neocollagenesis, and encourages neovascularization, thereby improving the elasticity and hydration of the vaginal mucosa [42]. RF generally achieves greater tissue penetration than lasers due to its lower frequency and longer wavelengths. The depth of RF penetration is influenced by the device configuration, with monopolar systems providing the deepest tissue coverage, while bipolar and multipolar systems—regardless of the presence of a cooling feature—are capable of delivering energy both externally to the vulva and internally to the vaginal mucosal epithelium and lamina propria [43]. Therefore, in our study, we demonstrated that monopolar capacitive radiofrequency was safe and effective in reducing the severity of GSM symptoms, as documented by the total VAS score (223 vs. 125; *p* < 0.001), and in ameliorating the sexual function, as evaluated by the FSFI-19 scale (22.9 vs. 38.6; *p* < 0.001). Specifically, the effect was more pronounced in the improvement of symptoms such as dyspareunia, vaginal dryness, and itching (*p* < 0.001), whereas the VAS scores for dysuria and burning did not show significant change. In addition, on medical evaluation, the appearance of the vaginal mucosa improved (VHI 13.5 vs. 16.5, *p* < 0.001), and, at the same time, most of the patients reported symptom relief (96% expressed a PGI score below 3). No evidence of burn injury or skin alteration was observed, and the treatment was considered painless for almost all the patients.

Our findings align with current literature on vaginal radiofrequency. In a previous study involving only 11 women reporting GSM symptoms, the same monopolar radiofrequency device was evaluated. One month after the end of treatment (T1), a significant reduction in symptoms was observed, with improvements reported in vaginal dryness (90.9%), pain during sexual activity (83.3%), vaginal itching (100%), burning (75%), and general pain (75%). The Vaginal Maturation Index (VMI) showed improvement in only half of the study population; however, sexual function, assessed using the Female Sexual Function Index (FSFI), and urinary symptoms, evaluated through the International Consultation on Incontinence Questionnaire-Short Form (ICIQ-SF), improved in 81.8% and 66.7% of participants, respectively, at T1 [44]. Vicariotto et al. similarly demonstrated the efficacy of a new low-energy dynamic quadripolar radiofrequency (DQRF) device in ameliorating the self-perceived sensation of vaginal laxity, as well as symptoms like dysuria/urinary incontinence and sexual dysfunction, in premenopausal women reporting vaginal introital looseness and in reducing the severity of GSM-related symptoms in postmenopausal women with a diagnosis of vaginal atrophy and dryness [45].

Furthermore, the mentioned results are consistent with those observed in several publications evaluating laser therapies. In a multicenter study, 645 patients suffering from GSM underwent three or four CO_2_ laser treatments, and statistically significant differences were observed in all evaluated parameters—dyspareunia (8.70 vs. 3.51; *p* < 0.0001), vaginal orifice pain (8.07 vs. 2.94; *p* < 0.0001), dryness/atrophy (8.30 vs. 2.97; *p* < 0.0001), itching (6.09 vs. 1.32; *p* < 0.0001), burning (6.12 vs. 1.78; *p* < 0.0001), and vaginal pH—when comparing pre-treatment and post-treatment values [46]. Similarly, in reference to the erbium YAG laser, Gambacciani at all. evaluated the long-term efficacy of the Fotona Smooth™ laser: three monthly sessions resulted in a significant reduction in VAS scores (*p* < 0.01) for both vaginal dryness and dyspareunia, along with a significant increase in VHI scores (*p* < 0.01), sustained up to 12 months after the final laser treatment. Additionally, vaginal erbium laser therapy improved mild to moderate stress urinary incontinence in 114 of 205 postmenopausal women enrolled [47].

Therefore, among energy-based treatments for GSM, both laser and radiofrequency technologies have shown promising efficacy in symptom relief and tissue regeneration. While both approaches involve relatively high costs, they differ in treatment protocols—laser therapy typically includes three sessions performed at intervals of 4–6 weeks [48], whereas radiofrequency requires weekly sessions. Laser treatments, particularly those using ablative technologies, are more frequently associated with adverse effects, such as pain, erythema, and edema, and tend to show a higher rate of intolerance. Nonetheless, lasers are supported by a wider body of clinical evidence, including long-term data extending up to 12 months [48], even in oncologic populations, who often present with more severe and persistent symptoms and have limited therapeutic options.

In this context, an Italian retrospective study focusing on cancer survivors included a cohort of 82 patients affected by breast cancer (BC) and VVA induced or worsened by adjuvant chemotherapy and/or hormonal therapy. After three cycles of fractional micro-ablative CO_2_ laser (one session every 30 to 40 days), significant differences in mean VAS scores were observed for sensitivity during sexual intercourse, vaginal dryness, itching/stinging, dyspareunia, and dysuria (*p* < 0.001 for all); bleeding and pain during probe insertion (*p* = 0.001); or movement (*p* = 0.011) [49]. Siliquini et al. confirmed the effectiveness of fractional CO_2_ laser in a cohort of 45 breast cancer survivors, though symptom improvement was slower compared to healthy controls, despite being sustained up to 12 months post-treatment [50].

While this is only a pilot study, our findings suggest that monopolar radiofrequency may represent a valid and promising alternative to existing treatment devices. The strengths of this study include its prospective design and comprehensive outcome assessment, addressing both objective improvements and the patients’ subjective perception of symptom relief, evaluated through multiple validated questionnaires. Moreover, a significant proportion of our cohort consisted of patients with hormone-dependent tumor histories (28 out of 48, 58.3%); however, at this stage of the study, we did not evaluate treatment response differences between the healthy and oncologic populations, which would be an interesting aspect to explore in future research. Nonetheless, several limitations must be acknowledged. The most significant limitation is the small sample size, which is consistent with the nature of a pilot study. To validate these results, future studies should involve larger populations, ideally within an RCT design. This would allow for the exclusion of placebo effects and provide a direct comparison between radiofrequency and established standard therapies. Another limitation is that the same clinicians who assessed the patients before treatment also conducted the evaluations during and after the intervention, which may introduce bias. Implementing a blinded assessment protocol would strengthen the reliability of outcome measures and reduce the risk of bias in both clinical and patient-reported outcomes. Furthermore, the follow-up period in our study was relatively short. Extending follow-up would help determine the long-term sustainability of the observed improvements and allow for further refinement of the radiofrequency protocol, including the optimal number of sessions, treatment intervals, and the potential need for maintenance therapy. These issues deserve careful exploration in future research.

## 5. Conclusions

Radiofrequency is an effective and well-tolerated outpatient procedure for vaginal functional restoration in the management of GSM and VVA. This technology appears to be minimally invasive, safe, and effective in alleviating clinical symptoms and enhancing patients’ quality of life. Consequently, it represents a valuable treatment option for patients who have contraindications to, do not respond to, or decline local estrogen therapy for GSM and VVA symptom relief. However, further research involving larger patient cohorts and extended follow-up is needed to define the optimal treatment protocol, including the ideal number of sessions required to maintain long-term benefits.

## Figures and Tables

**Table 1:** A comparative overview of the most common devices in GSM treatment.

Feature	CO_2_ Laser (Fractional Ablative)	Er: YAG Laser (Non-Ablative—SMOOTH™)	Radiofrequency (RF)
Energy Type	Light (laser—10,600 nm)	Light (laser—2940 nm)	Electromagnetic waves (0.3–10 MHz)
Mode of Action	Micro-ablative + thermal stimulation	Non-ablative deep thermal heating	Non-ablative, deep tissue heating via electrical current
Tissue Effect	Creates micro-columns of ablation + coagulation	Heats lamina propria without tissue removal	Heats deep tissues uniformly, no ablation
Depth of Penetration	~50–100 μm (superficial layers)	~200–500 μm (thermal effect in lamina propria)	Up to several millimeters (depending on device and settings)
Collagen Stimulation	Yes (via thermal injury)	Yes (via thermal stimulation)	Yes (via thermal stimulation)
Epithelial Integrity	Partially ablated (intact between micro-columns)	Fully preserved	Fully preserved
Healing Time	Short downtime (1–3 days)	No downtime	No downtime
Pain/Discomfort	Mild to moderate; may need topical anesthesia. Possible pain, burns, hyperpigmentation, or discharge	Minimal; usually no anesthesia	Painless, mild warming; no anesthesia needed
Number of Sessions	Typically 3 (4–6 weeks apart)	2–3 sessions (3–4 weeks apart)	3–5 sessions (1–4 weeks apart)
Clinical Effects	Increased thickness, elasticity, and hydration. Improved vaginal and urinary symptoms	Improved lubrication and elasticity. Regeneration without ablation	Improved moisture, elasticity, and mild urinary symptoms
Ideal For	Moderate–severe GSM; robust mucosa	Mild–moderate GSM; sensitive/thin mucosa	Mild–moderate GSM; non-invasive and device-dependent. Safe repeated use
Contraindications	Pregnancy, active infection, untreated cancer	Same	Same, plus pacemakers/metal implants

**Table 2:** Population baseline characteristics. Continuous data are reported as mean (SD). Non-continuous data are reported as absolute (relative) frequency.

Population Characteristics	Value
Age (Years)	53.9 (10.0)
Multiparous (%)	27 (56.3%)
Previous pelvic surgery (%)	26 (54.2%)
Oncology patients (%)	28 (58.3%)

**Table 3:** Comparison before (T0) and after (T1) radiofrequency treatment. FSFI-19: Female Sexual Function Index; VHI: Vaginal Health Index; PGI-I: Patients Global Impression of Improvement; and n/A: not applicable.

	T0	T1	*p*-Value
Total FSFI-19 score	22.9 (20.7)	38.6 (27.0)	*p* < 0.001
Desire	3.16 (1.3)	4.5 (1.8)	*p* < 0.001
Arousal	4.5 (4.8)	7.3 (5.4)	*p* < 0.001
Lubrication	4.3 (5.6)	7.4 (6.5)	*p* < 0.001
Orgasm	3.5 (4.0)	5.8 (5.1)	*p* < 0.001
Satisfaction	4.6 (3.7)	7.1 (4.6)	*p* < 0.001
Pain	3.0 (3.6)	5.2 (5.1)	*p* = 0.002
VHI score	13.5 (3.0)	16.5 (3.3)	*p* < 0.001
Total VAS score	223 (102.0)	125 (102.0)	*p* < 0.001
Dyspareunia	77.8 (33.4)	32.8 (32.8)	*p* < 0.001
Dryness	63.9 (42.9)	28.4 (32.2)	*p* < 0.001
Dysuria	5.2 (7.8)	5.8 (5.4)	*p* = 0.675
Vaginal burning	6.5 (9.4)	7.7 (7.3)	*p* = 0.422
Vaginal itching	75.0 (33.4)	42.2 (34.9)	*p* < 0.001
PGI-I	n/A	2.5 (0.7)	*p* < 0.001

## Data Availability

The data presented in this study are available upon request from the corresponding author.

## Abbreviations

The following abbreviations are used in this manuscript:

GSM	Genitourinary syndrome of menopause
VVA	Vulvovaginal atrophy
VRS	Vaginal relaxation syndrome
ET	Estrogen therapy
SERMs	Selective estrogen receptor modulators
DHEA	Dehydroepiandrosterone (Prasterone)
Er: YAG	Erbium-doped yttrium aluminum garnet (laser)
RF	Radiofrequency
VHI	Vaginal Health Index
VAS	Visual Analog Scale
FSFI-19	Female Sexual Function Index (19-item)
PGI-I	Patient Global Impression of Improvement
SD	Standard deviation
CE	Ethics Committee
VMI	Vaginal Maturation Index
ICIQ-SF	International Consultation on Incontinence Questionnaire-Short Form
DQRF	Dynamic quadripolar radiofrequency
BC	Breast cancer
